# Supplementary material for: Bayesian algorithm to estimate position and activity of an orphan gamma source utilizing multiple detectors in a mobile gamma spectrometry system
Source: PLoS One. 2021 Jan 22;16(1):e0245440. doi: 10.1371/journal.pone.0245440 (PMC7822251; doi:10.1371/journal.pone.0245440)
Supplement: Attachment — Submitted filename: Reviewer_comments_PLOS_ONE.pdf [file pone.0245440.s002.pdf]

Review of the manuscript entitled "*Bayesian algorithm to estimate position and activity of an orphan source utilizing multiple detectors in a mobile gamma spectrometry system*" by Bukartas *et al.*, submitted for publication to PLOS ONE.

**General comment:**

In this paper the authors developed a Bayesian algorithm to estimate the position of a lost photon (or more specifically a gamma) source. They have performed an extensive experiment by employing multiple detectors in a vehicle to perform on-site measurements. The paper addressed an important issue in the field, considering the detrimental effect of ionizing radiation on the health of general public and also the security related issues that are associated with nuclear materials. The paper is well-written, interesting, and the methodology has been discussed very well. I only have couple of minor comments on this manuscript. Therefore, I recommend minor revision of the manuscript before being considered for publication in PLOS ONE.

**The minor comments:**

1) Considering the Co-60 and Cs-137 sources that were studied in the present work, consider adding "gamma" to the title of your manuscript. Something like "Bayesian algorithm to estimate position and activity of an orphan **gamma** source utilizing multiple detectors in a mobile gamma spectrometry system".

2) The present work well-described the process of finding a lost gamma (photon) source. I believe this setup (and/or the methodology) cannot be used for a lost neutron source. The detectors that one needs to employ for neutron spectrometry should be something like Bonner sphere spectrometry (BSS) or nested neutron spectrometry (NNS) devices. Please consider adding this as one of the limitations on the present work.

3) Make sure all the words are consistent throughout the manuscript. For example, "preset" and/or "pre-set". Please double check the entire paper for such issues.

4) In line 25, "Sometimes, the additional survey can be severely limited…", please explain more on this.

5) In line 53, photon fluence rate (i.e., varphi in Latex) symbol is not matching the one shown in Eq. (1), please correct it.

6) In line 55, authors introduced the branching ratio, please mention the specific decay mode that was considered for the current radioactive sources that were studied in this work.

7) In line 80, "detector(-ors)" change to "detector(s)". Please check for issues similar to this in the other parts of the manuscript.

8) In line 95, fix the brackets (use two square brackets) for range of angles for θ. There are similar issues in other parts, please find and fix, example line 192.

9) In line 105, "Here, $\dot{N}_{sum}$ expresses a count rate in a stationary…", shouldn't it be "average count rate". The word "average" is crucial considering the Poisson statistics that you have introduced in later parts.

10) In line 107, "Then, let's consider a situation with a single unknown ionizing radiation source of…" How can the lost source be unknown? Almost all the radioactive sources are logged in radiation labs almost everywhere around the world (to best of my knowledge), so if it is lost then the source is known, which makes the so called "*search and rescue*" mission rather easier. Please clarify.

11) Eq. (9), please introduce $\lambda_{ij}$ as the mean of Poisson distribution which will be set to the sum of average count rate from the source and background.

12) In line 134, please mentioned gamma peak energies for Co-60 and Cs-137.

13) Table 1, I would just like to confirm that these are the setup variations that you have set. In first glance, it was very misleading as I thought these are measured/calculated results for different distances which does not make physical sense. Please considering adding a point to this table so readers know that these are variations in the setup that you have set to perform a sensitivity study.

14) Table 2 shows the average background count rates for different types of detectors. Background counts are measured in absence of the source, why there is a source variation here? Is it due to different ROI that was set for these two sources? Please explain and make appropriate changes to the manuscript to avoid confusion.

15) Results that are shown in Fig. 4 are interesting, please explain the significance of these results. To me the angular variations for Cs-137 and Co-60 look very similar.

16) Please considering citing following relevant papers;
1. Monte Carlo studies on neutron interactions in radiobiological experiments. PLoS ONE, (2017), 12(7): e0181281.
2. Monte Carlo studies on photon interactions in radiobiological experiments. PLoS ONE, (2018) 13(3): e0193575.
3. An Overview of the Application of Pulsed Neutron Activation in Flow Measurements. Nuclear Technology. 2020 Jul 2;206(7):1086-94.
4. Medium-thickness-dependent proton dosimetry for radiobiological experiments. Scientific reports. 2019 Aug 9;9(1):1-7.
5. Realistic dosimetry for studies on biological responses to X-rays and γ-rays. Journal of Radiation Research. 2017 Sep 1.
6. A calibration method for realistic neutron dosimetry in radiobiological experiments assisted by MCNP simulation. Journal of radiation research. 2016 Sep 30;57(5):492-8.
7. Conversion coefficients for determination of dispersed photon dose during radiotherapy: NRUrad input code for MCNP. PloS one. 2017 Mar 31;12(3):e0174836.
8. Studies on unfolding energy spectra of neutrons using maximum-likelihood expectation–maximization method. Nuclear Science and Techniques. 2019 Sep 1;30(9):134.

17) Table 3, please consider adding the standard deviations next to the mean values, something like mean ± err, given err is the standard deviation.

18) In line 258, "…the burn-in of 10000 iterations." Does that mean discarding 10000 iterations at the initialization of the run? Please explain.

19) In line 297, I am confused with "ARDD" and "PRDD", what does the second "D" letter represent at the end of ARD and PRD? Please explain.

20) Figure 6 and 7, I cannot see anything in those figures, firstly they are too small and packed and secondly, they have very poor quality/resolution. I guess you have no strict length limits in PLOS ONE, therefore please plot these two figures larger and make sure they are not so packed, then I can go through them after you have submitted the revised version.

21) Table 4, please explain what does negative ARD values represent.

22) Please explain the results of Table 4 and 5 in the text, just showing these numerical results with no explanation will not be acceptable.

23) In line 312, "are reasonably close to the true values (majority of absolute ARD and PRD values are less than 50%)". I actually see some large deviations for ARD and lesser for PRD, please explain.

24) Fig. 9, please check the unit of counting efficiency, the current unit in Fig. 9 does not seem correct to me. Considering the Eq. (11), I assume you should get something like $m^2$, the units for individual variables are (considering SI for simplicity);
N number of counts: #
r actual distance: m
A activity: #/s
n branching ratio: unitless
μ linear attenuation coefficient: $m^{-1}$
t measurement time: s

Please check and let me know if I misunderstood something here.

25) In line 347, "…Bayesian-based system might be affected by multiple additional factors." You have mentioned couple of factors here and I agree with you. I have done a full Monte Carlo particle transport calculation (note; this is different from MCMC method, my model simulates the realistic particle transport and tracks photons from the source and score their interaction with a detection volume that I have defined) for a Cs-137 source in ambient air at standard room temperature and pressure with no humidity or pollution. For simplicity the source assumed to be a mono-directional disk source emitting photon in only one direction (toward the detection volume), just to increase the interaction efficiency and make the problem simple. See Fig. 1 below for the photon energy distribution for different source-to-target distances,

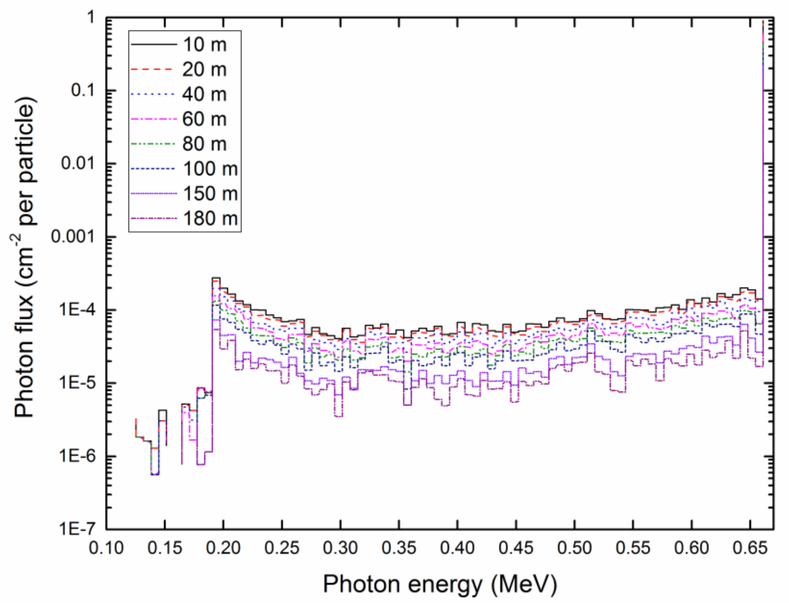


Fig. 1. Energy distribution of photons emitted from Cs-137 for different source-to-target distances.

In Fig. 2, total photon flux versus distance from detection volume (i.e., source-to-target distance) is shown;

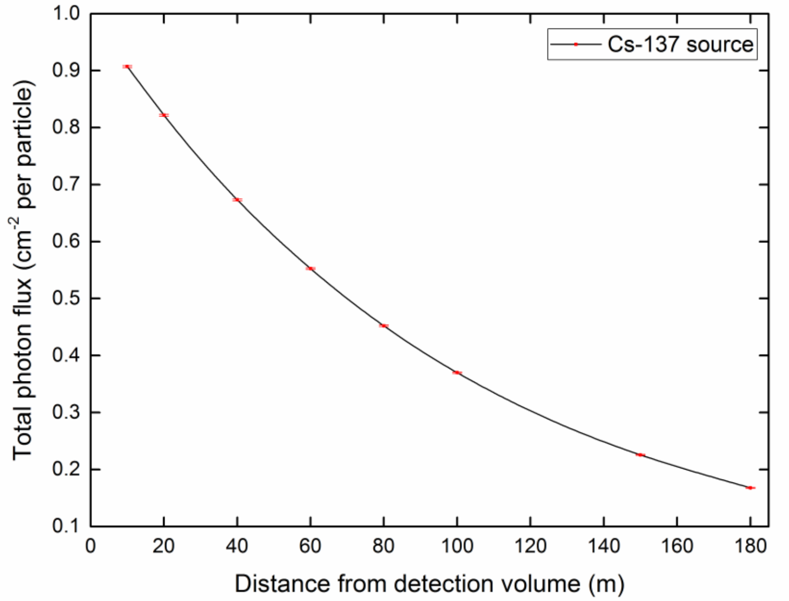


Fig. 2. Variation of total photon flux versus distance from detection volume for Cs-137 source.

It is evident that simply an air gap variation between the source and detector in the distance scale that you have studied can significantly alter or more precisely attenuate the beam of photons that are going to reach the detector. For the case of humidity in the ambient air this effect would be intensified. Considering these, please mention the factors that can have direct effect on the photon linear attenuation coefficients, such as chemical composition of medium, density of medium and

etc. In addition, one should note that these factors are not only affecting the Bayesian model, they directly effect the counts that were measured by the detectors, see Fig. 2 at which there is a large drop in the flux even for this “conservative” model that assumes all particles are being emitted toward the detection volume. For a point source, the values would be lower as particles would scatter in all direction.

26) In line 365, “when taking into account also the angular variation in the counting efficiency of the detectors used” Please re-phrase.

27) In line 367, “Additionally, the Bayesian inference would be performed faster due to less code in the Markov Chain Monte-Carlo loop”.
This statement is generally true. However, the performance of any computational methods/ algorithms depends on many factors. For example, the programming language that was used, code optimization, flow of variables, memory allocations, computer resources that were employed for its execution and etc. One can write a short piece of code that performs very inefficiently in a loop if it is not well optimized.

## End of my comments
