# Supplementary material for: Bayesian algorithm to estimate position and activity of an orphan gamma source utilizing multiple detectors in a mobile gamma spectrometry system
Source: PLoS One. 2021 Jan 22;16(1):e0245440. doi: 10.1371/journal.pone.0245440 (PMC7822251; doi:10.1371/journal.pone.0245440)
Supplement: Attachment — Submitted filename: Plos One Answer To Comments final AB.odt [file pone.0245440.s003.odt]

Reply to the comments:
The authors thank You for Your feedback. Hopefully, we have made all due corrections
to the article to make it more understandable for the readers. Following are the answers to the
questions.


1) Considering the Co-60 and Cs-137 sources that were studied in the present work, consider adding “gamma” to the title of your manuscript. Something like “Bayesian algorithm to estimate position and activity of an orphan gamma source utilizing multiple detectors in a mobile gamma spectrometry system”.

The title of the article changed according to the suggestion.

2) The present work well-described the process of finding a lost gamma (photon) source. I believe this setup (and/or the methodology) cannot be used for a lost neutron source. The detectors that one needs to employ for neutron spectrometry should be something like Bonner sphere spectrometry (BSS) or nested neutron spectrometry (NNS) devices. Please consider adding this as one of the limitations on the present work.

Currently, the presence of n-sources could already be detected indirectly by using gamma sensitive detectors, like it was displayed by Nilsson et al., 2015. If such a method would be used to detect the neutrons, then the Bayesian model has to be adapted of course, to correctly describe the physics of neutron detection in that particular situation.

Having a physical neutron detection model ready it would be only a matter of programming efficiency to implement it in the Bayesian algorithm. To confirm this, a separate, thorough investigation should be performed to properly test the applicability of the Bayesian method in neutron detection situations.  As the scope of the article is search of lost gamma sources, it probably should not be regarded as a limitation, but maybe more of a further possibility to explore the applicability of this apporach in different scenarios.

Nilsson, J.M.C., Finck, R.R., Rääf, C. Investigation and optimisation of mobile NaI(Tl) and 3He-based neutron detectors for finding point sources. Nuclear Instruments and Methods in Physics Research, Section A: Accelerators, Spectrometers, Detectors and Associated Equipment, Volume 786, 21 June 2015, Article number 57580, Pages 127-134

3) Make sure all the words are consistent throughout the manuscript. For example, “preset” and/or “pre-set”. Please double check the entire paper for such issues.

Corrected.

4) In line 25, “Sometimes, the additional survey can be severely limited...”, please explain more
on this.

As written in the text of the manuscript, there could be physical hindrances present (e.g. thick foliage, fallen trees, fences, other structures), preventing to access to the areas needed to estimate the position of the source. Additionally, these areas might be dangerous to enter due to e.g. too high activity of the source or suspected traps, set up by a perpetrator. In such and similar circumstances the ability to make estimations on position and activity of the source are of very high value for the decision makers.


5) In line 53, photon fluence rate (i.e., varphi in Latex) symbol is not matching the one shown in
Eq. (1), please correct it.

Amended.

6) In line 55, authors introduced the branching ratio, please mention the specific decay mode that was considered for the current radioactive sources that were studied in this work.

Text added for clarification.

7) In line 80, “detector(-ors)” change to “detector(s)”. Please check for issues similar to this in the other parts of the manuscript.

Corrected.

8) In line 95, fix the brackets (use two square brackets) for range of angles for . There are similar issues in other parts, please find and fix, example line 192.

This range, as you expressed, is a mathematical interval, and different brackets in intervals usually denote whether the specific end point of the interval is included or excluded. In the specific case of our manuscript, the angles are including 0 degrees (or 0 radians) but excluding 360 (or 2 pi radians) due to the overlap to avoid errors in the calculations. This notation is going to be kept as it is an international standard in mathematical notation covered in ISO 31-11.

9) In line 105, “Here, N sum  ̇ expresses a count rate in a stationary...”, shouldn’t it be “average count rate”. The word “average” is crucial considering the Poisson statistics that you have introduced in later parts.

Correct. The text in the manuscript was changed to clarify.

10) In line 107, “Then, let’s consider a situation with a single unknown ionizing radiation source of...” How can the lost source be unknown? Almost all the radioactive sources are logged in radiation labs almost everywhere around the world (to best of my knowledge), so if it is lost then the source is known, which makes the so called “search and rescue” mission rather easier. Please clarify.

Unfortunately, that is not the case in every situation. For example, in post-Soviet states during the changes of the government when the countries became independent, sometimes some parts or even complete journals of ionizing radiation source records were lost/destroyed. Because of that, there are still some ionizing radiation sources out of regulatory control present in those countries without known or even estimated location of some of them. 

In addition to that, however rare, serious radiological accidents due to lost sources have happened e.g. when a teletherapy source was left behind after a hospital has left the premises (accident in Goiânia, Brazil) or when unauthorized people removed a radioactive source from a radioactive waste repository (Accident in Tammiku, Estonia).

That aside, even if the information about the possible activity values of the source(s) is present, in a case where there are multiple sources scattered in an area, it would be of interest to estimate the activity of the sources from the first indicative measurements. Using the data acquired in these measurements, the position and activity of such sources could be identified even faster, without additional surveys employing e.g. hand-held equipment.

INTERNATIONAL ATOMIC ENERGY AGENCY, The Radiological Accident in Goiânia, , IAEA, Vienna (1988).

INTERNATIONAL ATOMIC ENERGY AGENCY, The Radiological Accident in Tammiku, , IAEA, Vienna (1998).


11) Eq. (9), please introduce ij as the mean of Poisson distribution which will be set to the sum of average count rate from the source and background.

Text changed.

12) In line 134, please mentioned gamma peak energies for Co-60 and Cs-137.

Added text for clarification.

13) Table 1, I would just like to confirm that these are the setup variations that you have set. In first glance, it was very misleading as I thought these are measured/calculated results for different distances which does not make physical sense. Please considering adding a point to this table so readers know that these are variations in the setup that you have set to perform a sensitivity study.

Precisely. The Table 1 is listed under Methods section, describing the methods and materials of the study, and as the caption of the table implies, that it covers specifications of distances from the source to the road side and activities for the experiment setups used in the field test. For the results of the study please refer to the “Results and Discussion” section.


14) Table 2 shows the average background count rates for different types of detectors. Background counts are measured in absence of the source, why there is a source variation here? Is it due to different ROI that was set for these two sources? Please explain and make appropriate changes to the manuscript to avoid confusion.

Yes, it is due to a different ROI. Another table was added (Table 2), Table 2 was changed (now Table 3) and some text was modified accordingly to avoid such confusion.

15) Results that are shown in Fig. 4 are interesting, please explain the significance of these results. To me the angular variations for Cs-137 and Co-60 look very similar.

As the total angular efficiency is a product of a reference efficiency (given in the Table 2) and the relative efficiency (displayed in the Fig. 4), there is no reason for the results to be drastically different. Because the reference efficiency of the detectors decrease with increasing energy as expected, thus, when calculating the total counting efficiency for a selected angle of incidence, the selected value of relative efficiency will be scaled down by the value of reference efficiency.


16) Please considering citing following relevant papers;
1. Monte Carlo studies on neutron interactions in radiobiological experiments. PLoS ONE,
(2017), 12(7): e0181281.
2. Monte Carlo studies on photon interactions in radiobiological experiments. PLoS ONE, (2018)
13(3): e0193575.
3. An Overview of the Application of Pulsed Neutron Activation in Flow Measurements.
Nuclear Technology. 2020 Jul 2;206(7):1086-94.
4. Medium-thickness-dependent proton dosimetry for radiobiological experiments. Scientific
reports. 2019 Aug 9;9(1):1-7.
5. Realistic dosimetry for studies on biological responses to X-rays and γ-rays. Journal of
Radiation Research. 2017 Sep 1.
6. A calibration method for realistic neutron dosimetry in radiobiological experiments assisted by
MCNP simulation. Journal of radiation research. 2016 Sep 30;57(5):492-8.
7. Conversion coefficients for determination of dispersed photon dose during radiotherapy:
NRUrad input code for MCNP. PloS one. 2017 Mar 31;12(3):e0174836.
8. Studies on unfolding energy spectra of neutrons using maximum-likelihood expectation–
maximization method. Nuclear Science and Techniques. 2019 Sep 1;30(9):134.


Thank you for providing so many articles for our consideration! It is a very nice collection of articles exploring radiobiological effects of ionizing radiation at different depths in the tissue, various methods estimating absorbed tissue dose and even calculation of neutron spectra.  Unfortunately, none of the articles you suggested are dealing with lost ionizing radiation sources, search for lost radiation sources in open areas in far geometries, full energy peak mobile gamma spectrometry using gamma radiation detectors or at the very least – Bayesian methods. Also, the MCMC methods utilized in the studies mentioned above are either custom written codes tailored to evaluate the neutron transport and resulting absorbed dose or already developed transport code like MCNP, which has no direct relevance in the scope of this paper. Despite that, we will have these articles in mind for future studies.


17) Table 3, please consider adding the standard deviations next to the mean values, something like mean ± err, given err is the standard deviation.


Added.


18) In line 258, “...the burn-in of 10000 iterations.” Does that mean discarding 10000 iterations at the initialization of the run? Please explain.

Because it usually takes some time (or iterations, to be more exact) for the Markov Chain Monte-Carlo algorithm to arrive to a high probability region and start sampling there, the first samples are usually discarded, as oftentimes they are not representing the distribution one tries to sample from.  Typically, in scientific literature dealing with Markov Chain Monte-Carlo algorithms, this action is denoted “letting the algorithm to burn-in” or “having a burn-in of … samples/iterations”.

Please, take a look at e.g.:
Yu, Z. Wei-qi, Q., Yuan-pei, S. Application of an adaptive MCMC method for the heat flux estimation. Inverse Problems in Science and Engineering, Volume 28, Issue 6, 2 June 2020, Pages 859-876
Jang, Y, Cohen, A.S. The Impact of Markov Chain Convergence on Estimation of Mixture IRT Model Parameters. Educational and Psychological Measurement, Volume 80, Issue 5, 1 October 2020, Pages 975-994
Hazra, I., Pandey, M.D., Jyrkama, M.I. Estimation of flow-accelerated corrosion rate in nuclear piping system. Journal of Nuclear Engineering and Radiation Science, Volume 6, Issue 1, 2020, Article number 011106

19) In line 297, I am confused with “ARDD” and “PRDD”, what does the second “D” letter represent at the end of ARD and PRD? Please explain.

Quite literally – “ARDD distribution” means “Activity Relative Deviation Distribution distribution”. ARD distributions has a scope of one particular experimental setup and ARDD distribution has a scope over all of the experimental setups. Thus, ARD distribution displays how well the algorithm could predict the activity for that particular experimental setup and ARDD distribution displays how well the algorithm was performing for all of the experimental setups on average.
Text in the manuscript changed for clarification.


20) Figure 6 and 7, I cannot see anything in those figures, firstly they are too small and packed and secondly, they have very poor quality/resolution. I guess you have no strict length limits in PLOS ONE, therefore please plot these two figures larger and make sure they are not so packed, then I can go through them after you have submitted the revised version.

We have been thinking for quite some time on the best way of visualizing the results. This approach was chosen as a compromise between the real-estate taken by the graphs and the simplicity of the approach. It is thus intended for Figures 7 and 8 to cover their whole respective separate A4 pages (at the figure size of 12 x 20 cm). The images are 1200 pixels wide and 2000 pixels high, translating to 254 DPI, resulting to file sizes of 7.3 MB. It was chosen to have the images at a bit lower, but still clear and acceptable resolution, than the required 300 DPI, as having the figure images with 300 and above DPI in tiff format results in file sizes above 10 MB, which is too high for submission according to the figure guidelines.  Despite that, the resolution is completely acceptable to portray the information in these simple graphs (see for example Fig. 2).

We have also noticed, that upon building the PDF for submission, the resolution of the images is reduced drastically for many of the figures. This raises some questions about the current methodology and/or some other technical aspects for the article submission system in PLOS ONE, as the figures that we are providing are of sufficient resolution and sufficiently clear to obtain and/or confirm the information needed. In Figs. 1 and 2 below we provide similar portions of screen captures of a part of Fig. 7 in the article obtained while building the PDF in PLOS ONE Editorial Manager and by compiling the PDF file from source materials using Latex and provided PLOS ONE Latex template respectively.

Fig. 1, Screen capture of how a part of a Fig. 7 is displayed in built-for-approval PDF version.

Fig. 2, Screen capture of the same part of the Fig. 7 from the article, when a PDF file is built using the source images using Latex (which are actually uploaded to the PLOS ONE)

Because we think that we are providing the figure images of sufficient quality and holding to as much of the guidelines as possible, we cannot do anything about this as the PDF is being built automatically, except to recommend for the reviewer to check the actual figure files submitted instead of the ones in the built PDF file. 

In the case of last resort, we can of course provide the full article in PDF version with all of the figures embedded inside with the images of correct resolutions (resulting in only 4.7 MB file) for the reviewers’ convenience.

21) Table 4, please explain what does negative ARD values represent.

Changed the text regarding the definition of ARD in the manuscript. As ARD is a relative difference expressed in percent, negative ARD values represent underestimation of the activity and positive – overestimation.


22) Please explain the results of Table 4 and 5 in the text, just showing these numerical results with no explanation will not be acceptable.

More in depth analysis of the results provided in the table was performed. Additional tables and figures were added to complement the requested analysis.


23) In line 312, “are reasonably close to the true values (majority of absolute ARD and PRD values are less than 50%)”. I actually see some large deviations for ARD and lesser for PRD, please explain.


	High median ARD and PRD values were obtained for data with relatively low signal-to-noise ratios (SNRs). Due to the way the experimental setups were arranged (which is discussed in detail in the Methods section), the biggest deviations were observed for experimental setups #4,8,12,16 and 20, because the sources were at their furthest for the given source activities. This resulted in low SNRs, which are displayed in the Tables 5 and 6 in the revised manuscript. Due to the low SNRs (signal from the source was comparable to the background) the height of the peak in the measurement time-series is almost undetectable, thus the algorithm tried to fit very distant source of extreme activity giving rise to almost undetectable peak in the measurement time-series. 
	In a figure below (Fig. 3,a) data fits are displayed for one of the estimations performed using the HPGe variation of the algorithm on one of the realizations of the 137Cs data from setup 8. It is clearly evident, that there is no peak visible in the measurement time-series after all, thus the algorithm has a hard time estimating the activity and position of the source given this data (majority of the estimations result in flat lines around the background level). 


a)b) Fig. 3, HPGe data fits for one of the 137Cs data realizations for experimental setup 8 (a) and setup 5 (b). Empty dots mark the simulated data, red line – expected mean number of counts in the detector based on the actual location and activity of the source and green lines display the fits of the data throughout the MCMC iterations. Dashed lines mark the 2.5% and 97.5% quantiles of the Poisson distribution for expected number of counts, given the true parameters.

Comparing the graph in Fig. 3, a, to a fit obtained for the same variation of the algorithm and source, but bringing the source closer (experimental setup 5, fits displayed in Fig. 3, b), we can clearly see the difference in the data and the performance of the algorithm. This is also evident in both the ARD and PRD tables and graphs. Despite that, none of the median values of ARDD or PRDD distributions (distributions of medians of ARD and PRD distributions throughout all of the experimental setups, which are displayed in the bottom of Tables 5 and 6) are above 50% of absolute relative deviation, thus displaying that the majority of the estimations lies within that 50% of the absolute relative deviation indeed. 
	In a situation, when searching for a lost ionizing radiation source, reasonably good estimations are within an order of magnitude of the actual activity of the source. There is not a lot of practical difference in how the a situation with a lost radioactive source should be handled by the decision makers if the activity of the source is e.g. 1 or 5 MBq, despite the 500%  difference. Thus, 50% relative difference between the estimated and actual activity values is really a reasonably good result.


24) Fig. 9, please check the unit of counting efficiency, the current unit in Fig. 9 does not seem correct to me. Considering the Eq. (11), I assume you should get something like m2, the units for individual variables are (considering SI for simplicity);
N number of counts: #
r actual distance: m
A activity: #/s
n branching ratio: unitless
μ linear attenuation coefficient: m-1
t measurement time: s
Please check and let me know if I misunderstood something here.

No, everything is correct – the total counting efficiency of the detector is expressed in m2.

We have calibrated the detector response as a product of two efficiencies – a reference efficiency (fixed efficiency of the detector with a source positioned in the plane of the detector at 90 degrees angle for a given radionuclide and indeed, expressed in m2) and a relative efficiency (a dimensionless number depending on the angle of incidence, that when multiplied with the reference efficiency, yields the total counting efficiency of the detector for that particular angle of incidence). As the reference efficiency is basically a simple scalar, the relative efficiencies were plotted to display how the efficiency of the detectors change depending on the angle of incidence. The text has been modified and a new table has been introduced to avoid confusion (Table 2)


25) In line 347, “...Bayesian-based system might be affected by multiple additional factors.” You have mentioned couple of factors here and I agree with you. I have done a full Monte Carlo particle transport calculation (note; this is different from MCMC method, my model simulates the realistic particle transport and tracks photons from the source and score their interaction with a detection volume that I have defined) for a Cs-137 source in ambient air at standard room temperature and pressure with no humidity or pollution. For simplicity the source assumed to be a mono-directional disk source emitting photon in only one direction (toward the detection volume), just to increase the interaction efficiency and make the problem simple. See Fig. 1 below for the photon energy distribution for different source-to-target distances,

Fig. 1. Energy distribution of photons emitted from Cs-137 for different source-to-target distances.
In Fig. 2, total photon flux versus distance from detection volume (i.e., source-to-target distance) is shown;

Fig. 2. Variation of total photon flux versus distance from detection volume for Cs-137 source.
It is evident that simply an air gap variation between the source and detector in the distance scale that you have studied can significantly alter or more precisely attenuate the beam of photons that are going to reach the detector. For the case of humidity in the ambient air this effect would be intensified. Considering these, please mention the factors that can have direct effect on the photon linear attenuation coefficients, such as chemical composition of medium, density of medium and etc. In addition, one should note that these factors are not only affecting the Bayesian model, they directly effect the counts that were measured by the detectors, see Fig. 2 at which there is a large drop in the flux even for this “conservative” model that assumes all particles are being emitted toward the detection volume. For a point source, the values would be lower as particles would scatter in all direction. 

	The attenuation of gamma photons in air is introduced in the Eq. 1 as a part of geometrical efficiency of the detector in the Theory section of the article, and taken in to account in the Bayesian model. Of course, the amount of the photons that are going to be attenuated is dependent on the atmospheric parameters like pressure, temperature and relative humidity. Factually – this is true, as the coefficient of linear attenuation of gamma particles in air is dependent on the density of air, which is in turn respectively dependent on the atmospheric parameters mentioned before.

To estimate the density of moist air, this equation can be used:
,
where, ρ is the density of the humid air (kg/m³), pd – partial pressure of dry air (Pa), Rd – specific gas constant for dry air, 287.058 J/(kg·K), T – temperature (K), pv – pressure of water vapor (Pa), Rv – specific gas constant for water vapor, 461.495 J/(kg·K), Md – molar mass of dry air, 0.0289654 kg/mol, Mv – molar mass of water vapor, 0.018016 kg/mol and R –R = universal gas constant, 8.314 J/(K·mol).

The vapor pressure of water may be calculated from the saturation vapor pressure and relative humidity. It is found by: 
formula,
where, pv is vapor pressure of water, ϕ is relative humidity and psat – saturation vapor pressure. The saturation vapor pressure of water at any given temperature is the vapor pressure when relative humidity is 100%.

One formula used to find the saturation vapor pressure expressed in Pascals is:
 formula,
where T is temperature in Celsius.
The partial pressure of dry air pd is found considering partial pressure, resulting in: 
formulap_{d} = p-p_{v} \,, where p simply denotes the observed absolute pressure. 
	Combining all of the equations mentioned above, it is possible to calculate the density of humid air, and then use it to obtain the values of gamma photon flux per emitted particle for ideal detector. Plugging in record pressure and temperature values in Sweden to obtain the most dense and most light atmosphere with relative humidities set to 0% and 100% the difference between the calculated fluxes at 100 metres for isotropically emitted Cs-137 photons is approximately 29%. The resulting graph is displayed below (Fig. 4). 

Fig. 4, Gamma photon flux per emitted particle for ideal detector for the most extreme logged weather parameters encountered in Sweden, combined to produce the most dense and most light air using relative humidity of 100% and 0% respectively.


It is important to stress again, that the chosen combinations of the record pressure and temperature with corresponding relative humidity values were chosen artificially. They were combined in such a way, that to produce the most dense and the most light air. Actual combinations of the parameters  were different for each parameter value. Also, relative humidity of 0% is basically impossible anywhere in the earth’s atmosphere, so such atmospheric property values are extremely unlikely to occur. So to obtain a graph resembling reality quite a bit more we took data from the atmosphere parameter measurements recorded within the last 4 months in the Malmö airport in southern Sweden, which is situated roughly 20 km east from city of Malmö. The data consists of hourly recorded atmospheric pressure, temperature and relative humidity values. Two sets of the values were chosen – one representing the atmospheric parameters with the highest recorded pressure and other with lowest. This time, actual combinations of the parameter values were chosen. The resulting graph calculated for two sets of the atmospheric values is displayed below (Fig. 5):

Fig. 5, Gamma photon flux per emitted particle for ideal detector for two of the atmospheric parameter combinations (combinations of parameters for most dense and most light air) measured in Malmö airport, Sweden, throughout the 4 most recent months.
#### Here, we can see that the there is approximately 5% difference between the calculated fluxes for the two different conditions at 100 metres from the source.
	Similarly, Finck R. in section I-44 of his PhD thesis has displayed that for actual extreme atmospheric property values in Sweden the atmospheric density variation is expected to be within the range of 1.1-1.4 kg/m3, resulting in a less than 10% difference between the fluxes.
	As the rate of change of the atmospheric conditions is usually quite a bit longer than the time-frame of the mobile gamma spectrometry surveys performed using ground vehicles, the atmospheric conditions (parameters) can be regarded as being relatively constant during the survey. Thus, practically, the Bayesian algorithm will be way more sensitive to irregular data distortions caused by e.g. gain drift in the NaI detectors or erroneous GPS timestamping. This renders e.g. variations in chemical composition of the atmosphere out of the scope of our study. 
Finck R., High Resolution Field Gamma Spectrometry and its Application to Problems in Evnironmental Radiology, PhD thesis, Departments of Radiation Physics, Malmö and Lund, Lund University, 1992.


26) In line 365, “when taking into account also the angular variation in the counting efficiency of the detectors used” Please re-phrase.

Clarified.

27) In line 367, “Additionally, the Bayesian inference would be performed faster due to less code in the Markov Chain Monte-Carlo loop”. This statement is generally true. However, the performance of any computational methods/ algorithms depends on many factors. For example, the programming language that was used, code optimization, flow of variables, memory allocations, computer resources that were employed for its execution and etc. One can write a short piece of code that performs very inefficiently in a loop if it is not well optimized.

	Indeed. But for the comparison to be valid there should be only one variable present at a time – irrespective of whether e.g. angular variations of efficiency in this exact instance are taken in to account or not. Others (e.g. programming language that was used, code optimization, flow of variables, memory allocations and computer resources) has to be the exactly the same or at the very least – somehow be taken in to account.
	To study the differences in performance of two non-identical programs written in two different programming languages (for example high-level programming language like R and a low-level language – C++) would be pretty much useless, as in the case of R and C++ comparison, the languages serve completely different purposes, thus the results could be influenced way more by the programming language than the actual differences in the code.
	Despite that, a more complex program (e.g. including more computational features) will inevitably require more computational resources, compared to a simpler program written in the same efficiency level, irrespective of the programming language it was written with...
